# Novel Model for Cascading Failure Based on Degree Strength and Its Application in Directed Gene Logic Networks

**DOI:** 10.1155/2018/8950794

**Published:** 2018-02-19

**Authors:** Yulin Zhang, Maoxian Zhao, Jionglong Su, Xiao Lu, Kebo Lv

**Affiliations:** ^1^College of Mathematics and Systems Science, Shandong University of Science and Technology, Qingdao, Shandong, China; ^2^Department of Mathematical Sciences, Xi'an Jiaotong-Liverpool University, Suzhou, Jiangsu 215123, China; ^3^College of Electrical Engineering and Automation, Shandong University of Science and Technology, Qingdao, Shandong, China; ^4^School of Mathematical Sciences, Ocean University of China, Qingdao 266100, China

## Abstract

A novel model for cascading failures in a directed logic network based on the degree strength at a node was proposed. The definitions of in-degree and out-degree strength of a node were initially reconsidered, and the load at a nonisolated node was proposed as the ratio of in-degree strength to out-degree strength of the node. The cascading failure model based on degree strength was applied to the logic network for three types of cancer including adenocarcinoma of lung, prostate cancer, and colon cancer based on their gene expression profiles. In order to highlight the differences between the three networks by the cascading failure mechanism, we used the largest-scale cascades and the cumulative cascade probability to depict the damage. It was found that the cascading failures caused by hubs are usually larger. Furthermore, the result shows that propagations against the networks were correlated with the structures motifs of connected logical doublets. Finally, some genes were selected based on cascading failure mechanism. We believe that these genes may be involved in the occurrence and development of three types of cancer.

## 1. Introduction

Over the past few decades, many scientists focused on the study of cascading failures in different networks, such as the electrical power networks [1–3], traffic networks [4, 5], Internet networks [6], social networks [7, 8], and even biological networks [9, 10]. The various models of cascading failures and their mechanisms, as well as their prevention, have been proposed. For instance, Motter and Lai [11] proposed a load-capacity cascading failure model and simulated an arbitrary power exponent of scale-free networks. The results showed that loads would redistribute among the nodes, and intentional attacks would lead to a cascade of overload failures, which could cause the entire part of the network to collapse. Wang and Xu [12] investigated cascading failures in coupled map lattices with different topologies and found that cascading failures are much easier to occur in small-world and scale-free coupled map lattices than in globally coupled map lattices. Crucitti et al. [13] presented a simple model for cascading failures based on the dynamical redistribution of the flow in the network, showing that the breakdown of a single node is sufficient to reduce the efficiency of the entire system if the node is among those with the largest load.

Recently, some researchers focused on the cascading failure mechanisms for directed networks. Fang et al. [14] proposed the cascading failure model in the context of directed complex networks. They used two attack strategies including minimum in-degree and the maximum out-degree attack strategy, which were compared with random attack strategy through simulations. Numerical results show that the cascading failure propagation in directed complex networks is highly dependent on the attack strategies and the directionality of the network. Jin et al. [15] built the load-capacity cascading failure model of the directed and weighted network. They applied the models to two typical real networks, namely, the Poisson distribution network and power law distribution network. Through simulation analyses, they concluded that the average weight and the average in-degree should be increased, respectively, for enhancing the resistibility of overloading and short-loading failures. Smart et al. [9] investigated the relationship between structure and robustness in the metabolic networks of* Staphylococcus aureus* and so on using a cascading failure model based on a topological flux balance criterion.

Despite this success, few studies have attempted to identify the cascading failure mechanism in a directed gene logic network. In this study, we investigate a load-capacity cascading failure model based on the degree strength of nodes and identify the influence of cascading failures on the gene logic networks. The directed network is constructed. The definitions of in-degree, out-degree, and degree strength are refined for different regulation types of second-order logical relationships. Then a novel algorithm for cascading failure based on load-capacity model is investigated. The load at a node is defined as the ratio of the in-degree strength to the out-degree strength of the node. The capacity of a node is the interval from the minimum load to the maximum load that the node can handle. By removing a particular gene node initially, the corresponding number of cascading failure nodes generated is noted. This process is repeated for each gene node in the network. The parameters, that is, the probability that a gene node will yield damage greater or equal to *d*, as well as the largest size ratio of cascading failure, are used to detect the relationship between network structure and robustness. Applying the model to gene expression profiles data for adenocarcinoma of lung, prostate cancer, and colon cancer, we find that hubs connected with other nodes by logical motif are more likely to break down. The study of cascading failure for gene networks may provide useful information underlying the biological mechanism of the formation and the development of cancers.

## 2. Methods

### 2.1. In-Degree and Out-Degree in the Logic Network

Bowers et al. [16, 17] proposed the logic analysis of phylogenetic profiles (LAPP) and demonstrated the benefits of identifying the relationships among gene triplets, as they have a greater likelihood of yielding the network organization of the interactions among gene triplets which forms the gene logical network. In fact, it can be considered as a weighted and directed graph that deciphers different logic interactions among gene node, including first-order and second-order logical relationships by the uncertainty coefficient at some thresholds (for details about the gene logical network, see Wang et al. [18] and Zhang et al. [19]).

**Figure 1:**
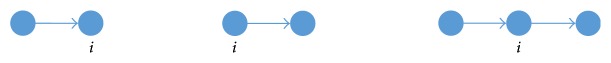
In-degrees and out-degrees for node *i* of all 1st-order logical relationships.

**Figure 2:**
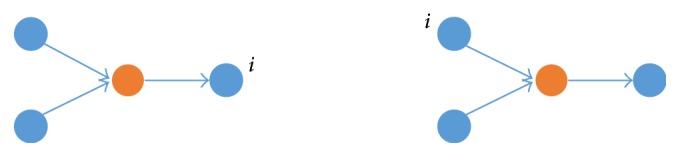
In-degree and out-degree for node *i* by some 2nd-order logical relationships.

In the first-order logical relationship, taking *A* → *C*, its uncertainty coefficient is defined as

(1) $$\begin{array}{c} U\left(\;C\;|\;A\;\right)=\frac{H\left(A\right)+H\left(C\right)-H\left(A,C\right)}{H\left(C\right)}, \end{array}$$

which measures the probability that gene *A* regulates gene *C*, where *H*(*B*) and *H*(*A*) are the Shannon entropies for vectors *A* and *B*, respectively, and *H*(*A*, *B*) is the joint entropy of *A* and *B*. This regulatory relationship is denoted as a weighted and directed edge.  Figure 1 gives three topologies for in-degree and out-degree for node *i* of 1st-order logical relationship. Obviously both the in-degree of *C* and the out-degree of *A* are increased by one for *A* → *C*. A second-order logical relationship as shown in Figure 2, for example, (*A*, *B*) → *C*, has an uncertainty coefficient denoted as *U*(*C*∣*f*_2_(*A*, *B*)) that measures the probability of existence of this second-order logical relationship. In this formula, *f*_2_ is the logical function. The uncertainty coefficient of (*A*, *B*) → *C* can be calculated by

(2) UC ∣ f2A,B=HC+Hf2A,B−HC ∣ f2A,BHC.

The second-order logic relationship (*A*, *B*) → *C* can be considered to be a directed edge with the weight *U*(*C*∣*f*_2_(*A*, *B*)). As in the LAPP method, all such gene triplets, with the corresponding *U* values, give rise to the gene logic networks further studied in our present work.

The definitions of the in-degree and out-degree need to be refined for different regulation types of second-order logical relationships, namely, AND, OR, and XOR. We propose these new definitions based on two principles: (1) the sum of the in-degree and that of the out-degree of all the nodes in a network are equal, and (2) the definition must be consistent with that of the degree and strength in first-order logical relationships.

Based on these two principles, the in-degree and out-degree of second-order logical relationships are defined as follows. If *k* regulates *C* (i.e., 1 appears *k* times), then the in-degree of *C* is increased by *k*/2. However, the out-degrees of *A* and *B* are determined by the proportion of their contributions to the second-order logical relationship. We can calculate the proportion based on the gene expression data particularly applied to gene networks in this research. Moreover, the second principle is meaningful only when it comes to the OR logic, as *A* and *B* regulate *C* simultaneously for AND logic.

For the XOR logic, we cannot determine how *A* and *B* regulate *C* (cooperatively or independently) merely from their gene expression profiles. For example, the specific algorithm to calculate the proportion of contribution from *A* to that from *B* is depicted by the third proper function *A*∨*B* → *C*. In a gene expression profile, components “1” and “0” denote the presence and the absence of the gene, respectively. An *n* × 3 matrix (*A*, *B*, *C*) with element 1 or 0 denote the gene expression profiles of genes *A*, *B*, and *C* expressed in columns, where *n* is the dimension of these vectors. Each row of the matrix is a three-dimensional vector, and each column is an *n*-dimensional vector. Let *n*_0_ be the frequency of row (1,1, 1), which indicates that both *A* and *B* activate *C*; let *n*_1_ be the frequency of row (1,0, 1) which indicates that only *A* activates *C*; and let *n*_2_ be the frequency of row (0,1, 1), which indicates that only *B* conducts the activation. The out-degree added to *A* by this second-order logic is (*n*_0_ + 2*n*_1_)/2(*n*_0_ + *n*_1_ + *n*_2_) times the total out-degree (i.e., the in-degree increment of *C*), and the out-degree distributed to *B* is (*n*_0_ + 2*n*_2_)/2(*n*_0_ + *n*_1_ + *n*_2_) times the total out-degree. Specifically, for the OR logic, the out-degree increments of both *A* and *B* are 3/4 according to the gene expression profiles of nodes in a network, and the in-degree increment of *C* is 3/2. For the AND logic, the out-degree increments of both *A* and *B* are 1/4 and the in-degree increment of *C* is 1/2. For XOR logic, the out-degree increments of both *A* and *B* are 1/2, and the in-degree increment of *C* is 1.  Table 1 lists the different types of logic relationship as well as their corresponding in-degrees and out-degrees.

### 2.2. Model of Cascading Failure for the Logic Network

**Definition 1 (in-degree strength and out-degree strength of a node).**

Suppose that there are *n*_1_ nodes regulating node *i* only by the first-order logical relationship. Therefore, the in-degree strength of node *i* is defined as in_*i*_ = ∑_*j*=1_^*n*_1_^*U*_*j*→*i*_, where *U*_*j*→*i*_ denotes the uncertainty coefficient of gene node *j* controlling gene node *i*. On the contrary, if node *i* is the source gene node regulating *n*_2_ other nodes by the first-order logical relationship, then the out-degree strength of node *i* can be defined as out_*i*_ = ∑_*k*=1_^*n*_2_^*U*_*i*→*k*_. Considering logical triplets, if node *i* is the target node of node *k*_*j*1_ and *k*_*j*2_ just by *n* second-order logical relationships, then the in-degree strength of node *i* is defined as

(3) ini=∑j=1nriUkj1,kj2→i+∑j=1n1−riUkj1,kj2→i=∑j=1nUkj1,kj2→i.

On the contrary, if node *i* and other nodes commonly regulate node *k* only by *m* second-order logical relationships, then the out-degree strength of node *i* is defined as out_*i*_ = ∑_*j*=1_^*m*^*r*_*i*_*U*_(*i*, *j*)→*k*_, where *r*_*i*_ corresponds to the types of second-order logic shown in Table 1. Finally, the total in-degree strength (out-degree strength) of node *i* is the sum of all in-degree strength (out-degree strength) of node *i* generating from both first-order logical relationships and second-order logical relationships.

**Definition 2 (load at a node).**

For a nonisolated node *i*, its load *l*_*i*_ can be defined in terms of its local information as the ratio of the in-degree strength to the out-degree strength. Specifically, if the in-degree strength of node *i* is equal to zero and the out-degree strength of node *i* is out_*i*_ > 0, then its load *l*_*i*_ = 0/out_*i*_ = 0. If the in-degree strength of node *i* is equal to in_*i*_ > 0 and the out-degree strength of node *i* is zero, then its load *l*_*i*_ = in_*i*_/0 = +*∞*. If the in-degree strength of node *i* is equal to in_*i*_ > 0 and also the out-degree strength of node *i* is out_*i*_ > 0, then its load *l*_*i*_ = in_*i*_/out_*i*_ = *k* > 0.

**Definition 3 (capacity of a node).**

Two capacities in node *i* are defined: for node *i*, the lower limit of capacity is *C*_*i*_ = (1 − *α*)*l*_*i*_ and the upper limit of capacity is *D*_*i*_ = (1 − *α*)*l*_*i*_, where parameter 0 < *α* < 1. Three cases are presented as follows: if *l*_*i*_ = 0, then *C*_*i*_ = *D*_*i*_ = 0, and the interval [*C*_*i*_, *D*_*i*_] shrinks to a point. If *l*_*i*_ = +*∞*, then *C*_*i*_ = *D*_*i*_ = +*∞*, and we consider that the interval [*C*_*i*_, *D*_*i*_] becomes +*∞*. If 0 < *l*_*i*_ < +*∞*, it forms a real interval [*C*_*i*_, *D*_*i*_] from the minimum load to the maximum load which the node can handle.

When all the nodes are active, the network operates in a free-flow state [11]. However, the removal of a node may cause the loads in other particular nodes to be redistributed to other components. The redistribution may cause the load to increase or decrease beyond the range of its initial capacity interval. In particular, the load may decrease from the positive value to 0 or increase to +*∞*. Thus, the corresponding nodes would collapse. As a result, subsequent failures would occur. Although it may stop after a few steps, it may also propagate and shut down a considerable fraction of the whole network. The cascading failure model depending on degree strength (D-SCFM) and the mechanism and the relationship between structure and stable are studied to control cascading failure against the gene logic network.

Let *G* = (*V*, *E*, *U*) be a logic network with the gene node set *V*, the directed edge set *E*, and the edge weighted set *U*. Suppose the logic network does not have multiple edges and self-loops. On the basis of the above-mentioned definitions and symbols, we propose an algorithm as follows.

*Input*. Initial matrix of the logic network *G* = (*V*, *E*, *U*).

**Step 1 .**

Initially select a node *j*, and then calculate its load *L*_*j*_^0^ and capacity *C*_*j*_^0^ = (1 − *α*)*L*_*j*_^0^, *D*_*j*_^0^ = (1 − *α*)*L*_*j*_^0^.

**Step 2 .**

Delete node *j* and its incident edges (both incoming and outgoing edges).

**Step 3 .**

Calculate the current load of remaining node and compare it with the initial capacity. Then delete any node which fails along with each of remaining edges.

**Step 4 .**

Repeat Step 3 until the failure will not happen.

## 3. Results

The real gene expression data are all downloaded from the Gene Expression Omnibus (CEO). All databases were based on the Gene Chip Human Genome U133A. The lung normal group was recorded as the control group I and the lung adenocarcinoma as the experimental group I. The prostate normal group was recorded as the control group II and the prostate cancer as the experimental group II. Similarly, the colon normal group was recorded as control group III and the colon cancer as the experimental group III. The specific situation is shown in Table 2. Furthermore, by using the Console Expression Software provided by Affymetrix Company, we obtain their value, *p* value, and corresponding *P*-*M*-*A* values, where *P* represents Present (expression), *A* represents Absent (not expressed), and *M* represents Margin. The *p* value in the database is recorded as 1, and the values of *A* and *M* are all recorded as 0.

However, there are few samples with too many genes (beyond 20000 genes) in each data set. We shall choose significant difference genes between the control groups and the experimental groups for the three types, respectively. We select candidate genes on the Wilcoxon rank sum test [19] at the significance level 2 × 10^−8^ by the corresponding values. Finally, 60, 65, and 79 genes were filed out from initial data and finally their expression matrices were obtained where each row represents a gene and contains a binary string of 0's and 1's to indicate the presence or the absence of the gene (http://cise.sdust.edu.cn/labs/other/zhangyulin/2017/workingdata.rar).

Two thresholds, namely, first-order *t*_1_ and second-order threshold *t*_2_ are used to detect the connections among nodes in the gene logic networks. We obtain the structural features including the numbers of nodes and edges versus two thresholds in Table 3. The number and distribution of the two order logic types in the networks change with the thresholds. The degree of each node subsequently changes, as a result its in-degree strength and out-degree strength will also change. With the increasing of threshold, the average degree of network nodes decreases. We try to analyze the relationship between robustness for cascading failure and network structural features such as degree and network motif under some thresholds.

**Figure 3:**
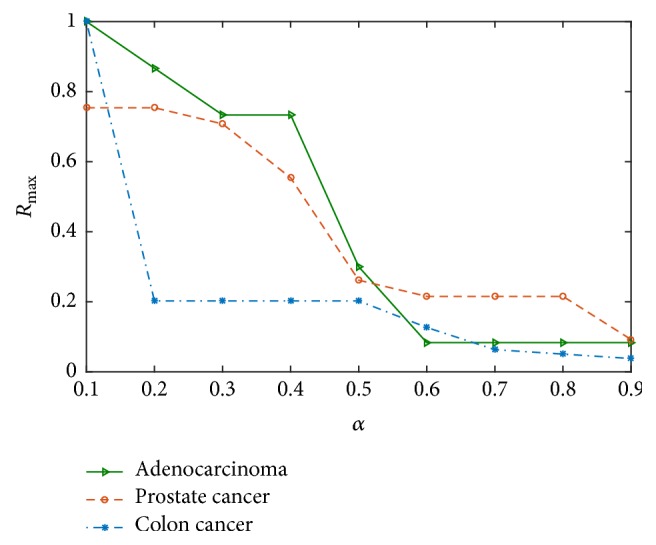
The change curves of the largest size ratio of cascading failure with increasing of capacity parameter.

By initially removing a gene node, failure cascades characterize the resultant cascade by its total number of other nodes deleted. After deleting node *i*, the failure of *s*_*i*_ nodes (including node *i*) and *d*_*i*_ = *s*_*i*_/*N* is an approximate indicator of network damage. The largest size ratio of cascading failure *R*_max_ = max⁡{*d*_*i*_, *i* = 1,2,…, *N*}. Let

(4) $$\begin{array}{c} \mathrm{sign}2\left(\;i\;\right)=\left\{\begin{array}{cc} 1, & d_{i}\geq d\\ 0, & d_{i}<d^{\prime }, \end{array}\right. \end{array}$$

where *d* is a variable parameter. Then the cumulative probability of cascading failures is defined as *P*(*d* ≥ *d*) = ∑_*i*=1_^*n*^sign⁡2(*i*)/*N*, denoting the probability that the network's cascading failures *d*′ are larger than *d*. The structural parameters defined above are used to measure the relationship between the network structure and the robustness of a network when successive failures occur. We focus on the key nodes that cause large-scale cascading failures on the network, that is, the key failure nodes, which are related to the parameters such as first-order threshold *t*_1_, second-order threshold *t*_2_, and capacity parameter *α*. Firstly, the capacity parameter *α* plays an important role in maintaining robustness of the network. Let *α* be a value from 0.1 to 0.9 with increment of 0.1.  Figure 3 gives the change curves of largest size ratio of cascading failure versus the capacity parameter *α*. Obviously, the smaller capacity parameter is, the more easily logical network is to fail in cascading failure.

**Figure 4:**
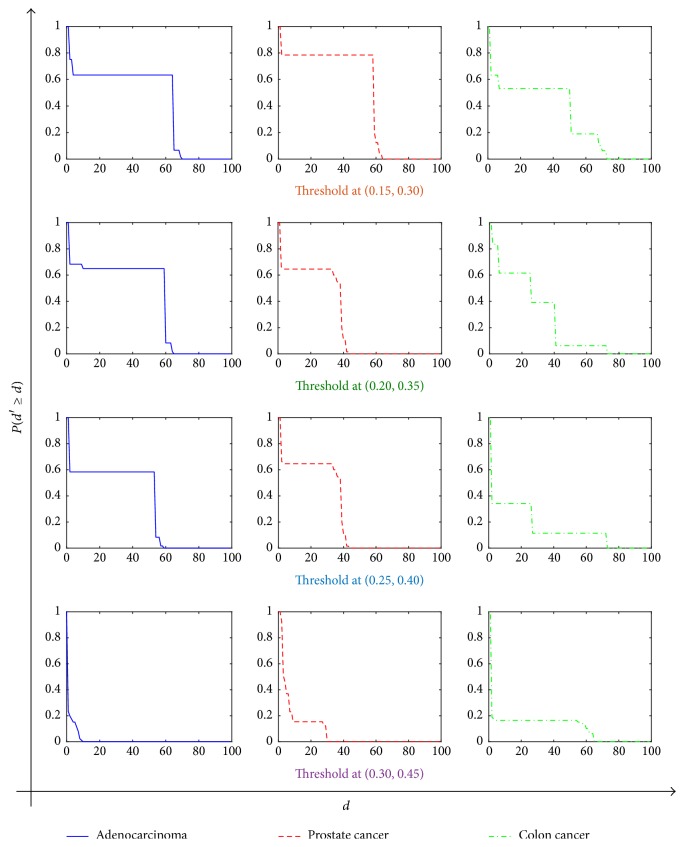
Plots of *P*(*d*′ ≥ *d*) versus *d* for gene networks with types of cancer under some thresholds.

If the thresholds are relatively small to zero, the connectivity of the network is very high. Not only is there no difference between the networks, but also the computational difficulty increases. While the thresholds are relatively large, lots of nodes in the network will be isolated. The selection of the thresholds is too large or too small not to conform to the practical biological significance. In the paper, four sets of thresholds at (0.15,0.30), (0.20,0.35), (0.25,0.40), and (0.30, 0.45) for three types of logical network are given to analyze the change of parameters for cascading failures. When we fix the parameter  *α* = 0.5, then the corresponding cumulative distribution curve *P*(*d*′ > *d*) for each type under the thresholds is shown in Figure 4. Obviously, with increasing values of *d*, *P*(*d*′ ≥ *d*) reduces to zero. The distributions *P*(*d*′ ≥ *d*) have a similar form for the types we studied: they are broad-tailed, indicating that most cascades are small, while some are quite large. These large failures represent lethal events, so that the behavior of *P*(*d*′ ≥ *d*) at large *d* is of special interest. In fact, with the increasing of thresholds, more and more isolated nodes and smaller connected branches appear in the network. The connectivity of the network is reduced, and the integrity of the network structure has been seriously compromised.

## 4. Conclusions

In our model, each node in the network is initially deleted and then cascading failure spread over the entire network. We try to obtain these nodes which can lead to the larger scale cascading failure. Removing a node initially, the failure of these nodes will lead to the failure of other nodes in the network. The four genes CDH1, MYC, SOS2, and CDKN1A are obtained from the prostate cancer network. Similarly, five genes including TOP2A, REL, SHH, ROS1, and CHEK2 in colon cancer gene network and three genes including RBL1, MAPK9, and PIK3CA in adenocarcinoma of lung gene network are selected.  Table 4 lists the gene nodes causing larger size cascading failure under all thresholds, where their in-degrees and out-degrees are given. It can be found that the nodes that cause the large-scale successive failures of the network are those nodes with larger in-degree or out-degree. The nodes with larger degree are closely associated with other nodes. If they are deleted, the cascades spread throughout almost entire network. However, the nodes with larger degree do not necessarily lead to large-scale cascading failures which are determined by the coupling relationship between nodes such as logical motifs.

**Figure 5:**
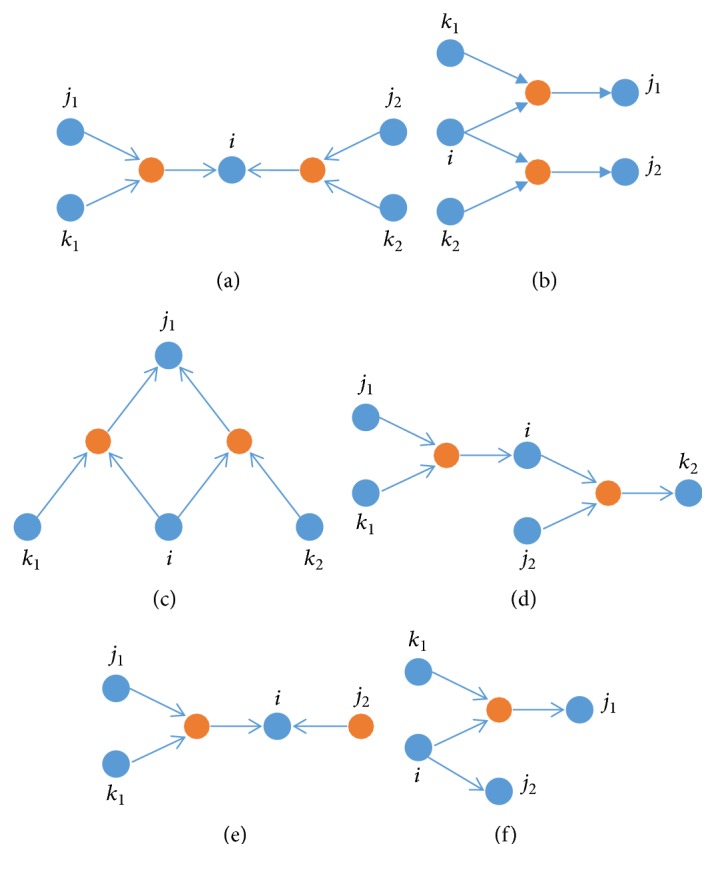
Six types of logic motif structures centered on node *i*.

The logic motifs are some doublets which are a combination of 2nd-order or 1st-order logical relationships with at least one common node. In Figure 5, (a), (b), (c), and (d) show all possible second-order logic doublets centered on node *i*. Nodes (e) and (f) in Figure 5 give another logic doublets centered on node *i*. These logic doublets are named according to the different positions of *i* as “both-in,” “both-out,” and “in-out” doublets. For example, (a) and (e) are “both-in” doublets for node *i*; therefore, node *i* has only incoming edge but no outgoing edge, so its load *l*_*i*_ = in_*i*_/0 = +*∞*. For (b), (c), and (f), node *i* has only outgoing edge but no incoming edge. So the in-degree strength of node *i* is equal to zero and the out-degree strength of node *i* is out_*i*_ > 0; then its load *l*_*i*_ = 0/out_*i*_ = 0. For (d), node *i* has both incoming and outgoing edge, so its in-degree and out-degree strengths are all greater than zero; hence, its load *l*_*i*_ > 0. If node *i* closely connected to other nodes by logical motifs is deleted initially, then it would cause any other nodes to break down easily.

## 5. Discussion

In the study, we look into the propagation of cascading failures in gene logic network occurring from initial failure using one by one deletion strategy. A new model based on load-capacity at nodes for cascading failure in the directed logic network is proposed. It attempts to explore the relationship between robustness and structure of the network. We apply the load-capacity cascading failure method based on degree strength to gene expression profiles data from the NCBI for three types of cancer gene networks including adenocarcinoma of lung, prostate cancer, and colon cancer. We find that if the hubs are deleted, it will cause larger cascading failure. As such these nodes are possibly related to the occurrence and development of three types of cancers.*Table 5 lists the genes and their gene annotations*.

Some genes have been confirmed in the literature associating with corresponding cancer. For example, Cherfas [20] found that gene CHEK2 is closely related to the occurrence and development of colon cancer. Cai et al. [21] detected the expression of SHH gene in 38 surgical resection of colon cancer. The aberrant state of the SHH signaling pathway may be involved in the development of colon cancer. Gene TOP2A encodes DNA topoisomerase, which can be used as a target for many anticancer drugs, and many of its variants are closely related to the development of resistance. The MYC gene is a regulator gene that codes for a transcription factor. It is located on chromosome 8 and believed to regulate expression of 15% of all genes through binding on Enhancer Box sequences and recruiting histone. This means that in addition to its role as a classical transcription factor, MYC also regulates the global chromatin structure by regulating histone acetylation both in gene-rich regions and at sites far from any known gene. Koh et al. [22] found MYC to be one of the top genes overexpressed in human prostate cancer tissues, as compared to matched normal-appearing prostate tissue.

Baldi et al. [23] found that the expression levels of RBL1, a protein similar to that encoded by the gene pRb2, were negatively related to the histological stage and metastasis of lung tumors. Therefore, gene RBL1 is a tumor suppressor gene of lung cancer. Gene PIK3CA encodes an alpha subunit of the phosphatidylinositol 3-kinase. Samuels and Velculescu [24] found high frequency variations of the PIK3CA gene in breast cancer and lung cancer. Most mutations are clustered in two locations in the PI3K helix or its catalytic role, and at least one hotspot mutation has increased kinase activity.

The paper proposed a load-capacity cascading failure model based on the degree strength of nodes and identified the influence of cascading failures on the gene logic networks based on their gene expression profiles. By numerical experiment, the parameters in the cascading failure model on the networks were analyzed to obtain the relationship between network structure such as degree and cascading failure. Finally, we obtained some gene nodes leading the larger scale cascading failure on the networks under the thresholds. These genes may play an important role in the development or metastasis of cancer. Due to the limited operation, Rank sum test is used to determine significant difference gene sets at a significant level firstly and this will inevitably lose some genes related to the specificity cancer. In addition, the specific biological significance of these genes still needs further validation by biologists.

## Figures and Tables

**Table 1:** Definition of in-degree and out-degree of 2nd-order logic.

Logic type	Out-degree of *A*	Out-degree of *B*	In-degree of *C*
*A*∩*B* → *C*	1/4	1/2	1/2
$\overset{-}{A}\cup \overset{-}{B}\to C$	3/4	3/4	3/2
*A* ∪ *B* → *C*	3/4	3/4	3/2
$\overset{-}{A}\cap \overset{-}{B}\to C$	1/4	1/4	1/2
$A\cap \overset{-}{B}\to C$	1/4	1/4	1/2
$\overset{-}{A}\cap B\to C$	1/4	1/4	1/2
$\overset{-}{A}\cup B\to C$	3/4	3/4	3/2
$A\cup \overset{-}{B}\to C$	3/4	3/4	3/2
*A*↔*B* → *C*	1/2	1/2	1
$\overset{-}{A↔B}\to C$	1/2	1/2	1

**Table 2:** Detailed list of data source.

Type	Group	Platform	Database	Value type	Sample size
Adenocarcinoma of lung	Control group I	GPL96	GSE12667	Value, *p* value *P*-*M*-*A*	73
	Experimental group I				68

Prostate cancer	Control group II	GPL8300	GSE6919	Value, *p* value *P*-*M*-*A*	50
	Experimental group II				43

Colon cancer	Control group III	GPL570	GDS3630	Value, *p* value *P*-*M*-*A*	40
	Experimental group III		GSE2109		39

**Table 3:** Network structure features for different cancer with change of thresholds.

Types	Thresholds	Number of nodes	Number of edges	Number of 1st logic types	Number of 2nd logic types	Average degree
Adenocarcinoma of lung	(0.15,0.30)	60	1008	92	916	25.21
	(0.20,0.35)	60	332	60	272	9.56
	(0.25,0.40)	60	297	42	255	7.60
	(0.30,0.45)	60	112	18	94	4.75

Prostate cancer	(0.15,0.30)	65	773	68	705	20.2
	(0.20,0.35)	65	255	29	226	6.71
	(0.25,0.40)	65	109	13	96	4.11
	(0.30,0.45)	65	50	12	38	3.28

Colon cancer	(0.15,0.30)	79	2543	94	2499	34.05
	(0.20,0.35)	79	1301	40	1261	32.23
	(0.25,0.40)	79	549	24	525	13.13
	(0.30,0.45)	79	324	15	309	7.77

**Table 4:** Degrees and *R*_max_ of some gene nodes in logical networks with three types of cancer under all the thresholds.

Threshold	RBL1			MAPK9			PIK3CA		
(0.15,0.30)	35%	165	44.75	25%	185.5	12	23.33%	58	9.5
(0.20,0.35)	31.7%	111	53.75	25%	74	31.75	20%	39	6.5
(0.25,0.40)	30%	63.5	22	16.7%	42.5	17.25	16.7%	34.5	5.5
(0.30,0.45)	15%	44	11.25	10%	26.5	8.75	8.33%	18	10.25

Threshold	CDH1			MYC			SOS2		

(0.15,0.30)	64.61%	125	35.5	50.77%	104	25.5	30.77%	76	31.25
(0.20,0.35)	50.70%	62	16.75	21.53%	58	10	16.92%	43	11.25
(0.25,0.40)	26.15%	16	7.75	18.46%	14	7.75	13.84%	12	6.5
(0.30,0.45)	12.31%	13	3.25	9.23%	8	5.25	9.23%	7	3.75

Threshold	CDKN1A			TOP2A			REL		

(0.15,0.30)	23.08%	49.5	17.5	88.6%	1239	22.75	32.91%	163	11.5
(0.20,0.35)	15.38%	26.5	14.25	53.16%	123.5	27.75	48.10%	60	45
(0.25,0.40)	15.38%	6.5	8.75	20.25%	41.5	14.75	20.25%	42.5	12.25
(0.30,0.45)	7.69%	5	2	13.92%	34.5	13.5	13.92%	31.5	9.5

Threshold	SHH			CHEK2			ROS1		

(0.15,0.30)	27.84%	89.5	41	27.84%	140.5	17	25.31%	69	14.5
(0.20,0.35)	45.57%	69	30.25	45.57%	68.5	27.5	25.32%	46.5	21.75
(0.25,0.40)	18.99%	41	4.75	13.92%	16	19.5	12.66%	16.5	8.75
(0.30,0.45)	11.39%	16.5	9.75	11.39%	13.5	5.75	7.59%	11.5	3.75

**Table 5:** Gene annotations selected by D-SCFM.

	Gene	Probe ID	Gene annotation
(1)	RBL1	1555003_at	Human RB transcriptional corepressor like 1
(2)	MAPK9	203218_at	Human mitogen-activated protein kinase 9
(3)	PIK3CA	204369_at	Human phosphatidylinositol-4,5-bisphosphate 3-kinase catalytic subunit alpha
(4)	CDH1	201130_s_at	Human cadherin 1 and related to the regulation of cell division
(5)	MYC	202431_s_at	Human v-myc avian myelocytomatosis viral oncogene homolog
(6)	SOS2	211665_s_at	Human SOS Ras/Rho guanine nucleotide exchange factor 2
(7)	CDKN1A	1555186_at	Human encoding an effective cyclin dependent kinase inhibitor
(8)	TOP2A	201292_at	Human topoisomerase (DNA) II alpha
(9)	REL	206035_at	Human REL proto-oncogene, NF-kB subunit
(10)	SHH	207586_at	Human sonic hedgehog and encoding SHH protein
(11)	CHEK2	210416_s_at	Human checkpoint kinase-2
(12)	ROS1	244363_at	Human ROS proto-oncogene 1, receptor tyrosine kinase
